# Virology, epidemiology, immunology and vaccine development of SARS-CoV-2, update after nine months of pandemic

**DOI:** 10.1016/j.biologicals.2020.11.003

**Published:** 2021-01

**Authors:** Marc Baay, Bruno Lina, Arnaud Fontanet, Arnaud Marchant, Melanie Saville, Philippe Sabot, Joris Vandeputte, Pieter Neels

**Affiliations:** a P95 Epidemiology & Pharmacovigilance, Leuven, Belgium; b University Claude Bernard Lyon, VirPath Research Laboratory, Lyon, France; c Department of Global Health, Emerging Diseases Epidemiology Unit, Institut Pasteur, Paris, France; d Institute for Medical Immunology, Université libre de Bruxelles, Brussels, Belgium; e Vaccine R&D, CEPI, London, United Kingdom; f International Alliance for Biological Standardization – IABS, Geneva, Switzerland

**Keywords:** SARS-CoV-2, Virology, Epidemiology, Immunology, Vaccine

## Abstract

This International Alliance for Biological Standardization COVID-19 webinar was organized to provide an update on the virology, epidemiology and immunology of, and the vaccine development for SARS-CoV-2, none months after COVID-19 was declared a public health emergency of international concern. It brought together a broad range of international stakeholders, including academia, regulators, funders and industry, with a considerable delegation from low- and middle-income countries.

## Highlights

• Recombination probably led to a virus that acquired human-to-human transmissibility.
• Initially, SARS-CoV-2 infection in the second wave was mistakenly interpreted as more benign.
• Neutralizing antibodies likely contribute to control of SARS-CoV-2 disease.
• A mucosal immune response (IgA in nasal mucus or saliva) may be a useful correlate of protection.
• COVAX aims to deliver 2 billion doses of vaccine by the end of 2021, to all the countries around the world.

## 1 Introduction

The International Alliance for Biological Standardization (IABS,^1^ https://www.iabs.org) is devoted to the scientific and medical advancement of biologicals, by facilitating communication among those who develop, produce and regulate biological products for human and animal health. Towards this end, IABS organized a second webinar on Covid-19 to update stakeholders from all continents, including more than 200 participants. The webinar consisted of four presentations, followed by ample time for discussion between speakers and participants.

## 2 Virology

Bruno Lina, professor of Virology at the University Claude Bernard Lyon and Director of the research laboratory VirPath, France, highlighted the different stages the virus went through during its emergence, the evolution of the viral genome, and whether these changes had any impact on transmission. This respiratory virus was highly transmissible from human to human from the start. When the virus was grown on human epithelial cells, the virus replicated very well, indicating it was fully adapted to humans. However, as a new virus it had come from a reservoir, most likely bats. The two previous introductions of new viruses, SARS-CoV-1 and MERS-CoV, came from the civet cat and the camel respectively. But these viruses were not fully adapted to humans because the transmissibility of these viruses was not as good as that of SARS-CoV-2; although some cases of transmission took place, the outbreak of the viruses could be controlled by observing some very basic hygienic measures, in contrast to SARS-CoV-2.

When looking at the first SARS-CoV-2 genome that was available, collected at a very early stage of the epidemic in the Wuhan wholesale market, this virus was a member of the beta-coronaviruses and it was very close to a bat virus that had been discovered a couple of months before, with a similarity of more than 96%, whereas it was quite different from other coronaviruses. As bats generally do not transmit viruses directly to humans, initially it was suspected that the pangolin was involved, because in the Guangdong area several coronaviruses which were quite close to the SARS-CoV-2 had been detected in pangolins. But from the phylogenetic tree of these viruses it was clear that the pangolin viruses were much more diverse than the bat virus, suggesting another animal was involved in the transmission from bats to humans, but it has not been identified as yet which animal.

The first French virus, which was sequenced on the 8th of February, was very close to the virus from the Wuhan market. However, looking at the spike protein, which is involved in the attachment of the virus to its receptor on the human cell, there is a huge difference between the SARS-CoV-2 and the two previously emerging beta-coronaviruses (SARS and MERS), indicating it really is a new virus. Comparing the sequence of the spike protein of the SARS-CoV-2 and its bat ancestor, most of the structure of the protein is identical, except for the region which interacts with the human receptor where some significant changes have been observed. These changes could not have occurred in the bat virus because it would have prevented the virus from attaching to and growing in bat cells. Therefore, it is possible that through recombination with another virus, the bat virus acquired the capability of binding well to the human receptor and then subsequently was introduced to humans. From very early, the virus was able to replicate in the upper respiratory tract and to transmit readily from human to human.

Since the beginning of the epidemic, an enormous amount of sequence data has been generated and these data are shared on the GISAID database, which is an asset for the surveillance of the virus. From these data, three general groups were identified, G, V and S, based on spatial distribution. The G group has a substitution in the spike protein (D614G) and this substitution has a competitive advantage, which enabled the G group to overwhelm the other viruses in less than two months’ time, although it did not completely displace the other ones, which are still circulating in different places around the world. One theory is that the G viruses have a better fitness with a higher transmissibility rate, which is still under investigation.

The G group has now been divided in three subgroups: G, GR, and GH. Looking at the level of the protein that is interacting with the ACE2 receptor, another group of substitutions has recently been identified in regions close to the receptor binding site, of which the S477 is most interesting (S477I/S477 N), with a possible enhanced capacity to bind to the receptor.

Looking at the overall evolution of the virus based on genomic surveillance of SARS-CoV-2 in France, with virus sequences from the early stage of the epidemic, March to June, there is very limited evolution of the virus. This is because the receptor binding site does not accept many sequence changes. In these viruses, changes occur at a maximum rate of two to three nucleotides per month. Moreover, there is also a very limited number of single nucleotide polymorphism (SNP) positions that can be impacted, fewer than 500, which means that the virus is highly stable, and, therefore not at all comparable to influenza.

In summary, the virus that emerged in December was perfectly fit, with all requirements for optimal dissemination in humans. The viral genome is highly stable, however recombination with other coronaviruses can occur, and deletions may also impact the virus. The introduction of the D614G substitution in February may have led to enhanced transmissibility. Similarly, the S477 N substitution may have led to increased affinity to the ACE2 receptor.

## 3 Epidemiology

Arnaud Fontanet, Director of the Department of Global Health, Head of the Emerging Diseases Epidemiology Unit, Institut Pasteur, France, shared the recent findings of studies with a focus on Europe, which are also relevant for other countries and regions. Mathematical modeling was done to construct the epidemic dynamics using data on hospitalization for COVID-19 in France. At the end of the lockdown in France, 5% of the population had been infected, with 10% for the two regions most impacted by the epidemic: the eastern part and the area around Paris. The basic reproductive number was estimated at 2.9, which means that to stop virus circulation, at least two-thirds of the population need to develop immunity, either through natural infection or vaccination [1].

To investigate if the lockdown had been effective in France to stop the virus from circulating and whether it was necessary to perform the lockdown at the national level as opposed to the regional level, hospital admission data were investigated by region for the 13 regions of France. It became clear that it was truly the lockdown which stopped the epidemic wave as all regions were already in a dynamic that would have resulted in a major local epidemic had a national lockdown not been performed. On March 17, 2020, the day of the lockdown, daily hospital admissions were indeed highest in the two affected regions but a surge in COVID-19 hospital admissions occurred at that time across all regions of metropolitan France [2]. The COVID-19 epidemic spread from the eastern to the western parts of France, crossing the daily hospitalization threshold of 1 per 100 000 inhabitants between March 10 (Grand-Est) and March 23, 2020 (Bretagne and Nouvelle-Aquitaine). Regardless of the time the epidemic started in the region, 12 out of 13 regions experienced a peak in daily hospital admissions on average 11 days (range 8–14 days) after the lockdown was implemented, which corresponds to the mean duration between infection and hospital admission for the patients experiencing severe forms of disease. Since the different regions were at different stages of the pandemic at the time the lockdown was implemented, the synchrony in regional peaks strongly suggests that the lockdown, rather than the natural course of the epidemic, explains the peak in hospital admissions [2].

Based on antibody testing, the proportion of the population that had signs of previous infection was fairly similar throughout Europe, with percentages ranging from 5% to 9% with population-based samples, with the exceptions of Finland where the percentage was much lower, at 1.6%, and somewhat higher percentages in bigger cities such as Geneva and Paris. Studies based on blood donors found percentages ranging from 2.6% to 7.1%, with an outlier in London, UK, where 14.8% of samples showed antibodies. These data show that in Europe, we are far from acquiring the herd immunity that we would need for the virus to stop circulating spontaneously [3], emphasizing the need for a vaccine.

To understand what happened during the summer, after the first wave, a heat map of Paris was constructed, looking at the age-specific SARS-CoV-2 incidence rate between May and October. May and June showed a very low incidence, whereas from the second week of August in the 20–29 years age group, the incidence rates exceed the threshold. From early October, the incidence rates started to increase in all age groups, reaching the older age groups. The heat map for Marseille, in the south of France, was very similar and it would be expected that this also goes for other European or American cities. Initially, the rising number of infections was not accompanied by a rise in hospitalization, because those young people did not get seriously ill. But by September and October, when the older age groups were reached, people started to be hospitalized. Although mathematical models predicted this pattern of low initial numbers of hospitalization based on age as a factor, the message in the media was that the virus had changed and that the disease would not be as severe as in the first wave. With a reproduction number of 1.4 there are few hospital admissions when the virus is at a very low level. But if the same dynamic continues, admissions to hospitals in general and intensive care in particular will become high, surpassing the threshold of number of beds available. Unfortunately, new measures were taken too late in France and other European countries.

The dynamic of the number of cases for several European countries suggests that initially there was an increase in infections when people came back from summer holidays, going back to school and jobs. However, this increase seemed to stabilize during the last two weeks of September. From the first week of October a very sharp increase in the number of cases was seen. This may have been caused by a change in the weather, which made people stay indoors, while at the same time the virus will more easily survive in colder conditions. At this point, to stop the spread of the virus, the only solution may be a second (partial) lockdown, as has been initiated in many European countries. As the virus will not go away by itself, it will be a very tough winter.

## 4 Immunology

Arnaud Marchant, Director of the Institute for Medical Immunology, Université libre de Bruxelles, Belgium, discussed three topics: the pathogenesis of COVID-19 and the role of innate and adaptive immune responses in this; possible cross-reactivity between other non-SARS coronaviruses and the role this could play in immunity to COVID-19; and finally the immunity induced by SARS-CoV-2 after infection and the implications this could have for people who have been infected by the virus previously.

SARS-CoV-2 infection can initiate a spectrum of clinical presentations, with systemic and organ inflammation, and infiltration of tissues by immune cells. Macrophages, neutralizing antibodies and T cells do not perform the way they should in the context of severe disease but result in an exacerbated response, with macrophages that are hyperactivated, producing cytokines that contribute to tissue damage and dysfunction [4]. Over the last months, a number of systems biology studies have been conducted essentially confirming this working model but looking more detailed at the cellular level and the molecular level. In the peripheral blood mononuclear cells of severe COVID-19 patients, reduced interferon-α production by plasmacytoid dendritic cells was observed [5]. On the other hand, enhanced plasma levels of inflammatory mediators were found, which correlated with disease severity. Single-cell transcriptomics revealed a lack of type I interferon (IFN) gene expression in peripheral blood immune cells of patients with severe COVID-19, and transient expression of IFN-stimulated genes [5]. Furthermore, unbiased screening identified SARS-CoV-2 proteins that antagonize IFN-I response via distinct mechanisms: suppression of interferon regulatory factor 3 phosphorylation, blocking of TANK-binding kinase 1 (TBK1) phosphorylation, and inhibition of IFN regulatory factor 3 nuclear translocation [6]. This SARS-CoV-2 evasion of IFN-I response may have an impact on viral transmission and pathogenesis. SARS-CoV-2 may be more efficient in IFN-I response evasion than SARS and MERS. The current working model is that under normal circumstances, with IFN-I production, the epithelial cells can control viral replication but when the virus blocks IFN-I production or the response is defective, this allows for viral replication and that will induce monocyte recruitment and inflammatory cytokine production, leading to disease. Considerable progress has been made in understanding the IFN-I response, including its spatiotemporal regulation and the prominent role of plasmacytoid dendritic cells, which are the main IFN-I-producing cells [7]. Well-timed IFN-I treatment and proposed strategies to boost IFN responses during the early stages of viral infection may be beneficial [7].

Two breakthrough studies have been published recently, supporting the goal for IFN-I in preventive life threatening severe COVID-19. The first study found an enrichment in rare variants predicted to be loss-of-function at the 13 human loci known to govern IFN-I immunity to influenza virus in patients with life-threatening COVID-19 pneumonia relative to subjects with asymptomatic or benign infection. Loss-of-function variants underlying autosomal-recessive or autosomal-dominant deficiencies were defined in a small subset of patients (3.5%), indicating that inborn errors of IFN-I immunity can underlie life-threatening COVID-19 pneumonia in patients with no prior severe infection [8]. Perhaps more intriguing is the discovery that about 10% of the patients with life-threatening COVID-19 have auto-antibodies against IFN-I [9]. The auto-antibodies were shown to neutralize the ability of IFN-I to block SARS-CoV-2 infection in vitro, and were not found in individuals with asymptomatic or mild SARS-CoV-2 infection. Data suggest that these patients had these auto-antibodies before the infection; they were not caused by the infection itself [9]. Both studies show that IFN-I plays a crucial role in the defense against SARS-CoV-2.

Another topic frequently discussed in terms of COVID-19 pathogenesis is the potential capacity of antibodies to enhance either infection or disease. When antibodies bind to viral particles, they can promote phagocytosis by macrophages, which leads to destruction of infected cells. In some viral infections like Dengue, these complexes can be internalized and lead to productive infection by macrophages. However, this is unlikely to happen with SARS-CoV-2 because macrophages cannot be productively infected by SARS-CoV-2.

Another possibility is enhancement of disease and this would involve the activation of innate immunity, including complement activation that promotes inflammatory responses, so the presence of antibodies could potentially exacerbate the inflammation itself. At present, there are no known clinical findings, immunological assays or biomarkers that can differentiate severe viral infection from immune-enhanced disease, whether by measuring antibodies, T cells or intrinsic host responses. In vitro systems and animal models do not predict the risk of antibody-dependent enhancement of disease, in part because protective and potentially detrimental antibody-mediated mechanisms are the same and designing animal models depends on understanding how antiviral host responses may become harmful in humans [10]. There is currently no evidence of antibody-dependent enhancement following SARS-CoV-2 infection, but this possibility should be kept in mind for studies of COVID-19 pathogenesis and for evaluation of vaccine candidates.

Antibody responses and the effector functions of antibodies are complex. This complexity calls for broad multiparametric assessment of antibody responses to SARS-CoV-2 infections. Profiling SARS-CoV-2-specific humoral responses in a cohort of hospitalized individuals, distinct antibody signatures resolved individuals with different outcomes. Although no differences in SARS-CoV-2-specific IgG levels were observed, spike-specific humoral responses were enriched among convalescent individuals, whereas functional antibody responses to the nucleocapsid were elevated in deceased individuals [11]. These data support the protective role of antibodies, especially when biased towards the spike protein, preventing fatal infection. In severe infections, T lymphocytes are depleted and dysfunctional with limited antiviral capacity. Severe COVID-19 is associated with lymphopenia, potentially exhausted T cells, interleukin-6-producing T cells, and reduced regulatory T cell functions [12,13].

Even if we understand better the pathogenesis of severe COVID-19, the relationship between the immune parameters and the risk factors that are associated with fatal outcome remain incompletely understood. A population cohort study was performed in the Western Cape, South Africa, using linked data from adults attending public sector health facilities [14]. Among more than 3 million patients (16% HIV positive), 22 308 were diagnosed with COVID-19, of whom 625 died. COVID-19 death was associated with male sex, increasing age, diabetes, hypertension and chronic kidney disease, as seen globally. Furthermore, HIV was associated with COVID-19 mortality with similar risks across strata of viral load and immunosuppression. Finally, current and previous tuberculosis were associated with COVID-19 death [14]. These data indicate that diverse populations may have different risk factors for severe COVID-19, involving distinct immunological pathways to need to be investigated.

Another point of interest is the role of exposure to non-SARS-CoV-2 viruses, and the induction of immunity to SARS-CoV-2. In all individuals convalescing from COVID-19, CD4 and CD8 T cells were found that recognized multiple regions of the N protein [15]. Patients who had recovered from SARS (the disease associated with SARS-CoV-1 infection) possessed long-lasting memory T cells that are reactive to the N protein of SARS-CoV 17 years after the outbreak of SARS in 2003; these T cells displayed robust cross-reactivity to the N protein of SARS-CoV-2. SARS-CoV-2-specific T cells were also detected in individuals with no history of SARS, COVID-19 or contact with individuals who had SARS and/or COVID-19. This suggests that infection with betacoronaviruses induces multi-specific and long-lasting T cell immunity against the structural N protein [15].

Looking at systemic and mucosal antibody responses in convalescent individuals who experienced varying disease severity, robust antibody responses to diverse SARS-CoV-2 antigens and evidence of elevated responses to endemic CoV were observed [16]. Assessment of antibody-mediated effector functions revealed an inverse correlation between systemic and mucosal neutralization activity and site-dependent differences in the isotype of neutralizing antibodies. Serum neutralization correlated with systemic anti-SARS-CoV-2 IgG and IgM response magnitude, while mucosal neutralization was associated with nasal SARS-CoV-2-specific IgA [16]. Convalescent patients, who have effectively stopped the infection, have increased responses to other coronaviruses, suggesting that some cross-reactivity occurs, and we may not all be equal in our response to SARS-CoV-2 because of previous contact with other coronaviruses.

Another parameter of pre-existing immunity that might be important is the state of the innate immune system and trained immunity; how the immune system responds to a broad range of pathogens and specifically the role that vaccines like BCG could have by promoting this trained immunity and these innate immune responses. A protective effect of BCG was shown in an interim analysis of the double-blind, randomized phase III ACTIVATE trial in elderly patients looking at the incidence of viral infections and respiratory infections over the period of one year [17]. BCG-vaccinated elderly patients had a two-fold decreased risk of viral infections, especially respiratory tract infection, without any adverse events, raising the question whether vaccination with BCG could protect against COVID-19 [17].

The third aspect of interest is the immunity induced by SARS-CoV-2 infection itself, which relates to the sporadic occurrence of recurrent infections. While the median time to seroconversion was nearly 12 days across all three isotypes tested, IgA and IgM antibodies against the spike protein were short-lived, with median times to seroreversion of 71 and 49 days respectively after symptom onset. In contrast, IgG responses decayed slowly through 90 days [18]. Of note, this study was performed on samples obtained from hospitalized patients, while it is known that the immune response in patients with mild symptoms or asymptomatic patients may have a shorter duration.

Concerning mucosal immunity, human challenge studies using coronaviruses showed that both circulating and local specific antibodies were associated with protection from infection and disease, but only specific IgA antibodies of either type appeared to shorten the period of virus shedding. Although total secretory IgA was significantly associated only with reduction of symptoms, total protein in nasal washings appeared to protect against infection also, indicating that IgA in nasal washings are correlates of protection against coronavirus [19]. Looking at the presence of IgA in the nasal wash of convalescent patients showed high levels of IgA in the nose and saliva of these patients and little difference between these two samples, suggesting that saliva could be an interesting site for sample collection [Sharma et al., unpublished data]. T lymphocytes were also induced by natural infection so overall immunity is induced by natural infection [20], but is it protective? To investigate this, studies are required to assess the risk of convalescent patients and uninfected controls over the coming winter period, to see whether natural immunity is protective and to find correlates of immunity.

Finally, an ongoing study is looking at effective transfer of SARS-CoV-2 antibodies to the newborn. Preliminary results show that those mothers that acquired SARS-CoV-2 infection very late, during the last part of pregnancy, are able to efficiently transfer humoral immunity to their baby, including antibodies promoting phagocytosis, complement activation, and NK-cell activation [Welba et al. unpublished data].

## 5 Vaccine development

Melanie Saville, Head of Vaccine R&D, CEPI (www.cepi.net), summarized the huge progress in vaccine development over the last six months. The WHO has put together a target product profile of COVID-19 vaccines: ideally, a vaccine would have 70% efficacy and would be a single dose, with a duration of protection of minimum one year. But given that this is a completely new disease, the minimum criteria are 50% efficacy, allowing for two-dose vaccines.

Currently more than 320 vaccines are being developed against SARS-CoV-2, based on a wide range of vaccine platforms. Five different categories of vaccines can be distinguished: vaccines using viral vectors, including adenovirus and measles vectors; the more innovative group of mRNA-based vaccines; DNA-based vaccines; protein-based vaccines with adjuvant; and the classic inactivated vaccines.

Normally in vaccine development, it would have taken multiple years to get to phase I trials. Within 2 weeks of publication of the sequence data, CEPI had announced three programmes to develop vaccine candidates against Sars-Cov-2. A number of clinical trials are already ongoing with 39 candidates, including 10 candidates in phase III. Eight of the vaccines in clinical testing are supported by the CEPI program, which will be made globally accessible through COVAX.

Initial results are available for several candidates. The candidate vaccine mRNA-1273 encodes the stabilized prefusion SARS-CoV-2 spike protein and was used in a phase 1, dose-escalation, open-label trial including 45 healthy adults, receiving two vaccinations, 28 days apart [21]. After the second vaccination, serum-neutralizing activity was detected in all participants evaluated. Solicited adverse events included fatigue, chills, headache, myalgia, and pain at the injection site. Systemic adverse events were more common after the second vaccination, particularly with the highest dose. Three participants in the highest dose group reported one or more severe adverse events [21].

NVX-CoV2373 is a recombinant SARS-CoV-2 nanoparticle vaccine composed of trimeric full-length SARS-CoV-2 spike glycoproteins and Matrix-M1 adjuvant [22]. A randomized, placebo-controlled, phase 1–2 trial evaluated the safety and immunogenicity of the rSARS-CoV-2 vaccine (in 5-μg and 25-μg doses, with or without Matrix-M1 adjuvant) in 131 healthy adults, receiving two intramuscular injections, 21 days apart. No serious or severe adverse events were noted. The addition of adjuvant resulted in enhanced immune responses, was antigen dose-sparing, and induced a T helper 1 (Th1) response. A neutralization response was induced that exceeded responses in convalescent serum from mostly symptomatic Covid-19 patients [22].

Finally, Ad26.COV2.S, a non-replicating adenovirus 26 based vector expressing the stabilized pre-fusion spike (S) protein of SARS-CoV-2 was administered as a single dose or as a two-dose schedule spaced by 56 days to adults and elderly [23]. Solicited local adverse events and systemic adverse events were less frequent in elderly. After a single dose, seroconversion was seen in 92% in adults and 83%–100% in the elderly, depending on dose. On day 14 post immunization, Th1 cytokine producing S-specific CD4^+^ T cell responses were measured in around 80% of participants, with no or very low Th2 responses, indicative of a Th1-skewed phenotype in both cohorts [23]. What can be seen quite consistently is that the vaccines generate binding antibody responses to the spike protein, with a varying degree of neutralizing antibody. The cellular immune response is Th1-oriented. Furthermore, some candidates are beginning to show data in the elderly population, which is one of the target populations for vaccination. Although generally, the immune response in the elderly is somewhat less than in adults, some candidates show strong data in an elderly population.

Some candidates are now getting very close to first efficacy readout. Towards licensure, there are three critical areas: the first is the efficacy of the vaccine, to have enough cases of SARS-CoV-2 infection, showing symptoms with five severe cases in the control group. The second piece that is critical is safety; the FDA have set strict rules for submission for emergency use authorization as to how much safety data is required, with a median follow up of two months post last vaccination. The third piece is controlled manufacturing at the scale needed for licensure. The first regulatory submission for emergency use authorization is expected before the end of the year. These submissions then have to be reviewed by the regulators and approved for use.

The various developers use different antigens from different strains, different assays and with different substrates, which makes it very difficult to compare the candidates at this point in time. CEPI is trying to make standardized and high-quality assays available to all developers. This includes developing antibody reference material and antibody standards that can be used in assays and developing a network of centralized laboratories that will allow for testing of neutralizing antibody and binding antibody as well as some of the cellular immunoassays. This is currently available for any developer who has a candidate in phase one or phase two and is provided free of charge [24].

To summarize, the reactogenicity profile is generally satisfactory, with some candidates showing increased reactogenicity after the second dose. No clinically relevant safety signals have been found to date. Although several trials were temporarily paused to evaluate safety, such pauses are not uncommon in clinical development to evaluate unexpected adverse events and demonstrate the effectiveness of the safety protocols that are in place. Long-term safety follow-up in ongoing Phase 3 trials is important, also to monitor for potential vaccine-mediated enhanced disease. Furthermore, a risk management strategy is needed to continue safety surveillance after licensure, which should be coordinated internationally.

Also, in the efficacy trials there are some nuances and differences between developers, e.g. in terms of statistical assumptions and sample sizes, although participant numbers are high in all studies.

The Access to COVID-19 Tools (ACT) accelerator, which is led by WHO, is looking at coordinating activities towards COVID-19 with pillars on diagnostics, therapeutics and vaccines. CEPI is a co-lead of the initiative for vaccines, where CEPI is responsible for development and manufacturing, GAVI is responsible for procurement and delivery of vaccines, and WHO is leading policy and vaccine allocation.

Under the umbrella of COVAX, CEPI, together with others, is has developed the largest and one of the most diverse portfolio of vaccines. The objective is to deliver 2 billion doses of vaccine by the end of 2021 through a coordinated approach. Those vaccines are to be distributed through a fair and equitable allocation process, developed by the WHO, to all the countries participating in COVAX. As of October 2020, 184 countries, representing over 90% of the global population, have joined COVAX to have access to vaccines for their citizens, including 92 low- and middle-income countries. For these countries fund-raising is ongoing for an advanced marketing commitment to ensure that these countries have access to vaccines at the same time as high-income countries.

In order to deliver 2 billion doses by the end of 2021 several successful candidates are needed. These candidates need to be produced by companies who can produce enough doses at an early stage to support clinical studies, scale up their processes to industrial levels before clinical trials begin, scale-out products in different countries to expand capacity, stockpile vaccines in bulk in anticipation of dose level definition, and repurpose facilities for successful products, if needed. Some candidates should be anticipated to fail during clinical development due to failing safety, efficacy, or manufacturing process requirements. To boost global manufacturing early, CEPI made a number of investments including agreements with Novavax, The University of Oxford and AstraZeneca, Clover Biopharmaceuticals, and University of Queensland and CSL to begin manufacturing millions of doses of their COVID-19 vaccine candidates, which – if proven safe and effective – were to be made available for globally fair allocation.

In summary, while there is rapid progress in vaccine development globally, with multiple efficacy studies ongoing around the world, manufacturing at scale and tech transfer is only just beginning. Hence, many challenges remain, especially for low resource settings. Two doses may be necessary, especially for the first vaccines. Current studies focus on adults and the elderly but there is a lack of data in other populations (e.g. children, immunosuppressed, pregnant women). Some mRNA-based candidates have ultra-cold chain requirements. Furthermore, the durability of the immune response and the need for a booster dose is currently unknown. Potentially, regulatory approvals will be needed in each country separately, for use in all countries. Every country may want a label in its own language, unless there is some pragmatism. Delivery and introduction will be cumbersome, especially in countries with limited infrastructure. And finally, upon introduction, strict pharmacovigilance will be necessary to continue to monitor safety, including the possibility of vaccine-mediated enhanced disease.

## 6 Discussion

Can mutations or modifications of the virus change the impact of the vaccines? Although new strains do emerge that are slightly different from the original Wuhan virus, no huge antigenic difference is seen and the strains in the vaccines will cross-neutralize. Surveillance will be critical to find new emerging strains.

The FDA have established some minimum requirements from a perspective of safety, with at least half of the population having at least two months safety follow up from the clinical trials, which is prudent under the current circumstances, because both the safety and the efficacy of the vaccine does need to be carefully evaluated. Moreover, the timeframe immediately after vaccination is the period in which you are likely to see safety issues. However, safety needs to be continuously monitored after emergency use authorization, to be able to find any safety issues in the longer term. Once the vaccine is more widely used, this can be done through pharmacovigilance systems and specific safety studies to evaluate rare events that can occur following vaccination.

The target product profile of the WHO is focusing on older age groups. Do the manufacturers foresee a particular emphasis on the different age groups? Although in the ideal situation vaccination would include all populations, we do know that certain populations are specifically at risk of severe disease and death. Even with the COVAX aim of 2 billion doses by the end of 2021, there will be a vaccine shortage initially, so the priority should be to first vaccinate those most at risk of severe disease. Hence, the first clinical trials are focusing on the elderly to ensure that there are robust data. Afterwards, other populations need to be studied, including children. We know that they get infected, but they rarely develop disease. This all needs to be part of the clinical development plan of the manufacturers. Investigation of the need for a pediatric formulation, which may be different from the adult formulation, also has to be part of the clinical development plan.

It is possible that none of the vaccine candidates would reach more than 50% effectiveness. Although everyone aims for vaccines that are as efficacious as possible, actually very few vaccines are 100% protective. There may always be some breakthrough cases, but 50% efficacy would already have a significant impact on disease and on the pandemic. Furthermore, the trial design for the phase three is aimed at case definitions of relatively mild disease. Quite often, a vaccine may have an efficacy of 50% against mild disease but much higher efficacy against severe disease. Therefore, it is essential to evaluate what the efficacy might be against more severe disease so that 50% efficacy endpoint could really have a significant impact on COVID-19, although this data is likely to come later.

Classically, before licensure the individual level is studied but modeling can be done to estimate the impact at a population level. The question is, what level of vaccination is needed to get to herd immunity for this respiratory virus? Animal data show that vaccines are preventing disease in the lung but some virus shedding from the upper respiratory tract is detected. So, as a direct effect, vaccines prevent disease in the individual. Indirectly, transmission is expected to be reduced by vaccination, but not completely eliminated. Modeling may help to look at how various vaccines could impact the population level and where herd immunity could be kicking in.

Concerning the lower frequency of infection, morbidity and mortality in developing countries, could the childhood vaccination with BCG play a role? The potential role of BCG is interesting but there is no conclusive answer yet; we should know hopefully soon. Several other possibilities have been proposed, the dominant one being age stratification and the age of the cases. Africa is a young continent and younger people suffer less disease, leading to overall lower mortality and incidence of severe disease. The state of the immune system at the time of infection is likely to play an important role. On the other hand, as shown in the study conducted in South Africa, HIV and active tuberculosis or previous tuberculosis could be risk factors for COVID-19 related death, which implies there are risk groups in countries in Sub-Saharan Africa.

One thing that is overlooked is what happens at the level of the mucosa. Most studies are looking at the systemic level; what happens if the virus gets into your nose is largely unknown.

What is the infectious dose? Does the dose relate to severity of disease? When human challenge studies are used as a model to test vaccines and therapeutics, the infectious dose in those studies may well give some insight. The relationship between infectious dose and severity of the disease has been observed and documented for other viral infections such as measles or varicella, where secondary cases in the family are more severe than the primary case, potentially related to a higher infectious dose for the secondary case.

Do all COVID-19 patients produce detectable antibodies? Looking back at studies where T cell responses were examined following mild or asymptomatic infection, no antibodies were found in a subset of patients, so it may be possible to have a T-cell response at a given time without having detectable antibodies. Whether those patients did not seroconvert or whether they had a transient immune response is very difficult to answer in cross-sectional studies and needs to be explored in prospective studies of the population.

How will post-marketing follow-up of efficacy and safety of the vaccines be organized? We have the efficacy trials that will get to licensure, but both post-licensure safety and efficacy studies will need to be performed. The risk management plan needs to plan those studies in the future, which is necessary from a regulatory perspective, but also needed to understand how effective a vaccine is in the real-world setting.

On safety, there seem to be tolerability issues, particularly for mRNA vaccines. Quite a lot of data have been gathered on the tolerability of those vaccines. They can be somewhat reactogenic, with local and systemic reactions in the days following vaccination. But the safety profile is similar to many other vaccines in terms of the reactogenicity.

How can it be explained that the elderly population, which has been still extensively vaccinated with BCG, is more at risk than the younger population, which was not vaccinated with BCG? BCG vaccination will not be the only main determinant of susceptibility to infection. Older age is associated with factors or determinants that impact disease outcome. The question is whether in the context of the susceptibility of older patients to severe disease, can this be changed by BCG vaccination?

Do immune imprinting issues occur by vaccines in contrast with wild type infection? An infection has a lasting effect on the immune system at large, however, it is unclear whether a natural infection will have long lasting imprinting effects. Major alterations of the immune system can be seen during the course of and soon after infection. But how long this will last, we don't know yet. In a study following up convalescent patients and controls over the winter season, SARS-CoV-2 infections and other infections caused by other respiratory viruses will be diagnosed. This will help to determine if previous SARS-CoV-2 infection changes susceptibility to infection by other viruses, which could be considered imprinting. As an infection generates massive alterations in the immune system, a vaccine, even a live attenuated vaccine, is less likely to invoke similar changes. If natural infection has negative effect on imprinting of the immune system, leading to increased susceptibility, then vaccination would be beneficial because the vaccine will prevent an infection that could have a negative effect.

Some vaccines may not have the efficacy that we would like to see. Human challenge studies, when carefully conducted, well-designed, and with appropriate protection of the participants may be the only way to answer some basic questions regarding why some vaccines are better than others and what could be correlates of protection. It is important to start thinking about them now in order to be ready if they are needed later. There are barriers to conducting human challenge trials. Firstly, it takes between six to nine months to have a fully acceptable CMC dossier for a challenge strain. Secondly, people will be quite reluctant to do challenge trials with COVID-19 as long as there is no accepted rescue therapy, as some people may die, even in the young target population for challenge trials. Nevertheless, some companies may be ready early next year to do the challenge trials. The benefit/risk balance of these challenge trials appears to be positive as people are dying from COVID-19 today anyway.

Can recovered patients be reinfected again and if so why? We do now have clear evidence that such recurrent infections do take place. But the number of cases is limited. Again, this can only be properly addressed in prospective studies. Recurrent infections could be more severe, exacerbated by pre-existing immunity, although the data are reassuring.

In conclusion, although SARS-CoV-2 shows limited evolution since its emergence, which is beneficial for vaccine development, recombination events with other human coronaviruses may occur. It is speculated that a recombination of the SARS-CoV-2 virus ancestor with another CoV occurred probably in the last trimester of 2019 leading to a virus that acquired human-to-human transmissibility.

Age-specific SARS-CoV-2 infection incidence rates in Paris, France, show that the incidence was low from mid-May to beginning of August, after which the incidence quickly rose in the 20-29-year age group. This slowly spread to older age groups, only affecting people aged 60 years and older a month later. This was mistakenly interpreted as more benign infection in the second wave since young individuals rarely experience severe forms of disease.

Defective production of or response to type I interferons and excessive production of inflammatory cytokines contribute to severe COVID-19 pathogenesis. Neutralizing antibodies likely contribute to control of SARS-CoV-2 disease and a mucosal immune response (development of IgA in nasal mucus or saliva) may be a useful correlate of protection. The durability and impact of infection-induced immunity is currently under investigation.

Of the more than 300 vaccine candidates, 39 are in clinical testing, with ten in phase 3 studies. Submission to regulatory authorities for emergency use authorization is expected before the end of the year 2020 for three vaccines. The objective of COVAX – an end to end partnership of Research and Development, manufacturing, procurement and fair allocation – is aiming to deliver 2 billion doses of vaccine by the end of 2021, to be distributed in a fair and equitable allocation process to all the countries around the world.

## Declaration of competing interest

The authors have no competing interests to declare.

## Footnotes

1 CEPI - Coalition for Epidemic Preparedness Innovations; CoV – coronavirus; IABS – International Alliance for Biological Standardization; ICU – intensive care unit; IFN – interferon; WHO – World Health Organization.

## References

[1] Salje H., Tran Kiem C., Lefrancq N., Courtejoie N., Bosetti P., Paireau J. (2020). Estimating the burden of SARS-CoV-2 in France. Science (New York, NY).

[2] Cauchemez S., Kiem C.T., Paireau J., Rolland P., Fontanet A. (2020). Lockdown impact on COVID-19 epidemics in regions across metropolitan France. Lancet.

[3] Fontanet A., Cauchemez S. (2020). COVID-19 herd immunity: where are we?. Nat Rev Immunol.

[4] Tay M.Z., Poh C.M., Rénia L., MacAry P.A., Ng L.F.P. (2020). The trinity of COVID-19: immunity, inflammation and intervention. Nat Rev Immunol.

[5] Arunachalam P.S., Wimmers F., Mok C.K.P., Perera R., Scott M., Hagan T. (2020). Systems biological assessment of immunity to mild versus severe COVID-19 infection in humans. Science (New York, NY).

[6] Xia H., Cao Z., Xie X., Zhang X., Chen J.Y., Wang H. (2020). Evasion of type I interferon by SARS-CoV-2. Cell Rep.

[7] Sa Ribero M., Jouvenet N., Dreux M., Nisole S. (2020). Interplay between SARS-CoV-2 and the type I interferon response. PLoS Pathog.

[8] Zhang Q., Bastard P., Liu Z., Le Pen J., Moncada-Velez M., Chen J. (2020). Inborn errors of type I IFN immunity in patients with life-threatening COVID-19.

[9] Bastard P., Rosen L.B., Zhang Q., Michailidis E., Hoffmann H.H., Zhang Y. (2020). Autoantibodies against type I IFNs in patients with life-threatening COVID-19.

[10] Arvin A.M., Fink K., Schmid M.A., Cathcart A., Spreafico R., Havenar-Daughton C. (2020). A perspective on potential antibody-dependent enhancement of SARS-CoV-2. Nature.

[11] Atyeo C., Fischinger S., Zohar T., Slein M.D., Burke J., Loos C. (2020). Distinct early serological signatures track with SARS-CoV-2 survival. Immunity.

[12] Vabret N., Britton G.J., Gruber C., Hegde S., Kim J., Kuksin M. (2020). Immunology of COVID-19: current state of the science. Immunity.

[13] Chen Z., John Wherry E. (2020). T cell responses in patients with COVID-19. Nat Rev Immunol.

[14] Boulle A., Davies M.A., Hussey H., Ismail M., Morden E., Vundle Z. (2020). Risk factors for COVID-19 death in a population cohort study from the Western Cape Province, South Africa. Clin Infect Dis.

[15] Le Bert N., Tan A.T., Kunasegaran K., Tham C.Y.L., Hafezi M., Chia A. (2020). SARS-CoV-2-specific T cell immunity in cases of COVID-19 and SARS, and uninfected controls. Nature.

[16] Butler S.E., Crowley A.R., Natarajan H., Xu S., Weiner J.A., Lee J. (2020). Features and functions of systemic and mucosal humoral immunity among SARS-CoV-2 convalescent individuals.

[17] Giamarellos-Bourboulis E.J., Tsilika M., Moorlag S., Antonakos N., Kotsaki A., Domínguez-Andrés J. (2020). Activate: randomized clinical trial of BCG vaccination against infection in the elderly. Cell.

[18] Iyer A.S., Jones F.K., Nodoushani A., Kelly M., Becker M., Slater D. (2020). Persistence and decay of human antibody responses to the receptor binding domain of SARS-CoV-2 spike protein in COVID-19 patients. Sci Immunol.

[19] Callow K.A. (1985). Effect of specific humoral immunity and some non-specific factors on resistance of volunteers to respiratory coronavirus infection. J Hyg.

[20] Sekine T., Perez-Potti A., Rivera-Ballesteros O., Strålin K., Gorin J.B., Olsson A. (2020). Robust T cell immunity in convalescent individuals with asymptomatic or mild COVID-19. Cell.

[21] Jackson L.A., Anderson E.J., Rouphael N.G., Roberts P.C., Makhene M., Coler R.N. (2020). An mRNA vaccine against SARS-CoV-2 - preliminary report. N Engl J Med.

[22] Keech C., Albert G., Cho I., Robertson A., Reed P., Neal S. (2020). Phase 1-2 trial of a SARS-CoV-2 recombinant spike protein nanoparticle vaccine. N Engl J Med.

[23] Sadoff J., Le Gars M., Shukarev G., Heerwegh D., Truyers C., de Groot A.M. (2020). Safety and immunogenicity of the Ad26.COV2.S COVID-19 vaccine candidate: interim results of a phase 1/2a, double-blind, randomized, placebo-controlled trial.

[24] CEPI CEPI establishes global network of laboratories to centralise assessment of COVID-19 vaccine candidates. https://cepi.net/news_cepi/cepi-establishes-global-network-of-laboratories-to-centralise-assessment-of-covid-19-vaccine-candidates/.

